# Calomel in Heart Cases

**Published:** 1910-04-23

**Authors:** 


### CALOMEL IN HEART CASES.

Historically speaking, calomel as a diuretic, especially in cases of cardiac œdema, is a very old remedy. Boerhaave employed it in the seventeenth century and remarks in one of his works: "Cure may be produced by means of small doses of sweet quicksilver in cases of ascites which are associated with a dropsy of the whole body, since the urine is thereby caused to flow in very large quantities." The celebrated Dublin physician, William Stokes, was a strong advocate of calomel in these cases. Nevertheless of recent years this teaching of our predecessors is to some extent falling into the background on account of the introduction of digitalis.

An extensive investigation into the effect of calomel in heart disease has been carried out under the direction of Professor Lenhartz by Dr. Simons, who records the full account of the diuresis produced in twenty-four consecutive cases. The summary of his conclusions is that even when all the other better-known diuretics have been employed in vain, a considerable diuresis was produced in nineteen of the patients, whilst in nine of them the increase in the urine outflow amounted to 4,000 c.c. per diem. The greatest individual amounts of urine that were observed in any one case were 8,500 c.c. on the fifth day, and in another case 6,200 c.c. on the fifth day; in one child of eleven years of age no less than 3,800 c.c. were passed on the fifth day.

The most marked diuresis nearly always occurred upon the fifth day, and it seldom happened that much effect was observed until calomel had been given for four days; it is important, therefore, to repeat the calomel each day for at least five days, and not to be deterred from repeating it simply because there is no diuresis at first. The doses employed varied according to circumstances, but in many cases not less than two grains were given three times a day.

After the administration is stopped the diuresis often continues for a couple of days longer, and then the amount of urine passed gradually falls again. In every instance in which it is purposed to employ calomel in this way, however, it is important to pay very particular attention to the cleansing of the mouth, especially when there is any affection of the teeth. After every meal some mild antiseptic mouth-wash should be employed for rinsing out the mouth, and a tooth-brush should be used with an antiseptic tooth-powder or paste. Fairly strong potassium permanganate is a good mouth-wash, provided the solution is not so deeply tinted as to cause discoloration of the teeth. If the state of the mouth is not particularly attended to in this way there is a considerable liability to stomatitis; on the other hand, with proper care, no stomatitis ensues from the large doses mentioned above.

It is most important whenever there is albuminuria to examine the centrifugalised deposit of the urine microscopically to be sure that no kidney epithelial cells or renal-tube casts are present before the mercurial treatment is begun.

